
JOURNAL INFORMATION
==============================
NLM Title Abbreviation: Behav Neurol
Journal ID: Behav Neurol
NLM Journal ID: 8914585
Journal ID: BN

Behavioural Neurology

ISSN: 0953-4180
EISSN: 1875-8584
Publisher: Wiley

ARTICLE INFORMATION
==============================
PMCID: PMC5469955
PMID: 18430981
DOI: 10.1155/2007/201512
Article ID: 201512
Article version: 1
Subjects: Clinical Note

Spatial Perseveration in Dementia with Lewy Bodies

Assal Frédéric 1 *
McNamara Patrick 2
1 Neurology Department of Clinical Neurosciences University Hospital-HUG Micheli-du-Crest 24 Geneva Switzerland
2 Neurology Boston University School of Medicine and VA New England Healthcare System 150 South Huntington Avenue Boston MA 02130 USA
*Frédéric Assal: frederic.assal@hcuge.ch
Print publication date: 2007
Electronic publication date: 2008 Mar 5
Volume: 18
Issue: 4
First page: 235
Last page: 236
Received 2008 Feb 18; Accepted 2008 Feb 18
Copyright: Copyright © 2007 Hindawi Publishing Corporation and the authors.
Copyright year: 2007
License: This is an open access article distributed under the Creative Commons Attribution License, which permits unrestricted use, distribution, and reproduction in any medium, provided the original work is properly cited.
License URL: https://creativecommons.org/licenses/by/3.0/

==============================
Behavioural Neurology 18 (2007) 235–236
IOS Press

Clinical Note

### Spatial perseveration in dementia with Lewy bodies

Frédéric Assal[a,*] and Patrick McNamara[b]
[a]*Neurology, Department of Clinical Neurosciences, University Hospital-HUG, Micheli-du-Crest 24, Geneva, Switzerland*
[b]*Neurology, Boston University School of Medicine and VA New England Healthcare System, 150 South Huntington Avenue, Boston, MA 02130, USA*

An 80 y.o. right-handed man presented with a left bradykinesia, cognitive fluctuations, unexplained falls, disrupted sleep, complex visual hallucinations and delusions evolving for 10 months. Instrumental activities of daily living were impaired for finances and shopping abilities. He was alert, oriented and very slow. On neurological exam, he had a positive glabellar response and left predominant akinetorigid syndrome. Mini Mental Status Examination was 20/30. The clock drawing test (CDT Fig. 1A), and subsequent writing (Fig. 1B), disclosed a peculiar type of perseveration that was apparently restricted to the compulsion to reproduce spatial representational schemas (draw a circle etc). Extensive neuropsychological testing demonstrated attentional, executive, visuospatial and episodic memory deficits. Moderate corticosubcortical atrophy was noted on MRI. Final diagnosis met all criteria for probable dementia with Lewy bodies (DLB) [1].

The CDT is quick to administer, relatively independent of culture, language and education, easy to score for research purposes [2], and valid as a screening tool for moderate to severe cognitive impairment in older adults [3]. Patients are asked to draw a clock-face, showing all the numbers, and the hands to set the clock to 10 minutes past 11 o'clock, then sometimes to copy a correctly drawn clock-face. Qualitative assessment addresses numerous cognitive functions such as comprehension and semantic knowledge, planning, visual memory, visuospatial abilities, motor programming and execution, numerical knowledge, abstract thinking, inhibition and concentration. Common errors in dementia include right-left confusion, spatial distortions, hemi-neglect, confusion about the concept of time, loss of planning, tendency to pull the minute hand towards "10" (stimulus bound) and perseverations.

The CDT has been used to differentiate DLB and Alzheimer's disease (AD) patients matched for severity of dementia. When DLB was diagnosed in patients with higher scores for drawing than the copy conditions, the CDT had 100% specificity, 100% positive predictive value, 73% negative predictive value but only 21% sensitivity [4]. These findings were challenged by another group who found this pattern in AD patients and concluded that better drawing was not specific for DLB [5]. Global quantitative measures of CDT performances were also the same between patients with AD, DLB or Parkinson's disease although qualitatively, the latter two made more planning errors than patients with AD [6]. None of these authors mentioned any perseveration of any type. In the literature, perseverations may be recurrent (repetition of a previous response to a subsequent stimulus), stuck-in-set (inappropriate maintenance of a category or framework of activity) or con-

*Corresponding author: Dr. Frédéric Assal, Neurology, Department of Clinical Neurosciences, University Hospital-HUG, Micheli-du-Crest 24, 1211 Geneva, Switzerland. Tel.: +41223728318; Fax: +41223728332; E-mail: frederic.assal@hcuge.ch.

ISSN 0953-4180/07/$17.00 © 2007 – IOS Press and the authors. All rights reserved

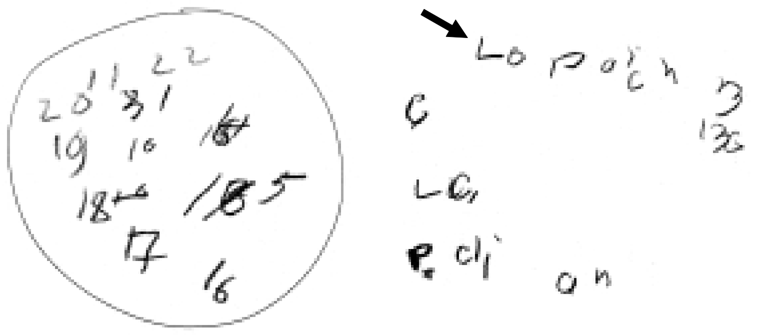

A B

Fig. 1. (A). Clock-drawing test: spatial disorganization, poor and slow planning, numerous stuck-in-set perseverations, loss of flexibility (writes 10 10 instead of placing the hands; (B). Sentence under dictation "the little cat drinks good milk" ("Le petit chat boit du bon lait"): peculiar recurrent perseveration or spatial perseveration on previous clock with letter reiteration and dysgraphia (arrow indicates the beginning of the sentence.

tinuous (abnormal prolongation of a current activity), and reflect impairment of distinctive neuroanatomical systems such as prefrontal and parietal association cortical networks, basal ganglia, and their connections [7, 8]. In unspecified dementias, perseverations are usually continuous or stuck-in-set [2]. The CDT of our patient not only demonstrated common errors found in DLB such as spatial disorganization, poor and slow planning, stuck-in-set perseverations, loss of flexibility but he also produced a dramatically new type of perseveral we call spatial perseveration. This particular type of recurrent perseveration of the subsequent dictated sentence "in a clock mode" constitutes a unique combination of both executive and visuospatial impairments that are well-established and prominent in DLB [1]. We acknowledge the limits of a single-case report in addressing this issue but this spatial perseveration constitutes unique neuropsychological evidence for a distinct processing in cognitive architecture that accesses spatial representations, and then makes them available for support output of action or behavioural sequences or guidance of action scripts (in this case 'draw a clock').

### References

[1] I.G. McKeith, D.W. Dickson, J. Lowe et al., Diagnosis and management of dementia with Lewy bodies: third report of the DLB Consortium, *Neurology* **65** (2005), 1863–1872.

[2] K.I. Schulman, Clock-drawing: is it the ideal cognitive screening test? *Int J Geriatr Psychiatry* **15** (2000), 548–561.

[3] Y. Nishiwaki, E. Breeze, L. Smeeth et al., Validity of the Clock-Drawing Test as a screening tool for cognitive impairment in the elderly, *Am J Epidemiol* **160** (2004), 797–807.

[4] K.K. Gnanalingham, E.J. Byrne and A. Thornton, Clock-face drawing to differentiate Lewy body and Alzheimer type dementia syndromes, *Lancet* **347** (1996), 696–697.

[5] G.R. Swanwick, RF. Coen, C.P. Maguire et al., Clock-face drawing to differentiate dementia syndrome, *Lancet* **347** (1996), 1115.

[6] D.A. Cahn-Weiner, K. Williams, J. Grace et al., Discrimination of Dementia With Lewy Bodies From Alzheimer Disease and Parkinson Disease Using the Clock Drawing Test, *Cogn Behav Neurol* **16** (2003), 85–92.

[7] J. Sandson and M.L. Albert, Perseveration in behavioral neurology, *Neurology* **37** (1987), 1736–1741.

[8] Y. Nagahama, T. Okina, H. Nabatame et al., The cerebral correlates of different types of perseveration in the Wisconsin Card Sorting Test, *J Neurol Neurosurg Psychiatry* **76** (2005), 169–175.
